# Supplementary material for: Itinerant topological magnons and spin excitons in twisted transition metal dichalcogenides: mapping electron topology to its spin counterpart
Source: Natl Sci Rev. 2025 Aug 23;13(4):nwaf354. doi: 10.1093/nsr/nwaf354 (PMC12878470; doi:10.1093/nsr/nwaf354)
Supplement: nwaf354_Supplemental_File [file nwaf354_supplemental_file.pdf]

Supplemental material for "Itinerant topological magnons and spin excitons in twisted transition metal dichalcogenides: Mapping electron topology to spin counterpart"

Wei-Tao Zhou, Zhao-Yang Dong, Zhao-Long Gu, and Jian-Xin Li

METHODS

Tight-binding model

To generate a tight-binding model and the corresponding Wannier basis in the subspace of our interest is to fix a gauge freedom at each momentum $\boldsymbol{k}$, which is a $U(1)$ phase for subspace containing only one band, a $U(1) \times SU(2)$ matrix for two bands and a $U(1) \times SU(N)$ matrix for N bands. In order for the tight-binding model to be a good one, the Wannier basis should be as localized as possible so that the hopping would fast vanish with the distance to present a compact tight-binding Hamiltonian by proper cut off (see Extended Data Fig. 5).

From continuum model the eigen state $\psi_{\mu\boldsymbol{k}}(r)$ has the following form

$$\psi_{\mu\boldsymbol{k}}(\boldsymbol{r}) = \sum_{\boldsymbol{G}} \sum_{l=b,t} a^{\boldsymbol{G}}_{\mu\boldsymbol{k}l} \chi^{\boldsymbol{G}}_{\boldsymbol{k}l}(\boldsymbol{r}) \tag{1}$$

where μ, l is the band and layer index, respectively. $\boldsymbol{G} = n_1\boldsymbol{g}_1 + n_2\boldsymbol{g}_2$, $\boldsymbol{g}_{1,2}$ is the reciprocal wave vector. $\boldsymbol{G}$ sums over reciprocal lattice sites in the cut off $|\boldsymbol{G}| \leq t|\boldsymbol{g}_1|(t = 3$ is often enough for convergence). In the plane-wave approximation, $\chi^{\boldsymbol{G}}_{\boldsymbol{k}l}(\boldsymbol{r}) = \eta_l e^{i(\boldsymbol{k}+\boldsymbol{G})\cdot\boldsymbol{r}}$ and $\eta_l = (1,0)^T$ or $(0,1)^T$ for bottom/top layer.

Instead of strictly obeying the maximally localized Wannier function criterion, we generate the Wannier states from a semiclassical point of view. As shown in Extended Data Fig.5**b**, $\Delta_{b,t}(\boldsymbol{r})$ reaches its maximum at XM/MX stacking region which serves as a quantum well to confine the electron. We can first perform a layer polarization $\varphi'_{n\boldsymbol{k}}(\boldsymbol{r}) = \psi_{\mu\boldsymbol{k}}(\boldsymbol{r})U'^{\dagger}_{\mu n}(\boldsymbol{k})$ to maximize $\langle\varphi'_{1\boldsymbol{k}}| P_b |\varphi'_{1\boldsymbol{k}}\rangle$ and $\langle\varphi'_{2\boldsymbol{k}}| P_t |\varphi'_{2\boldsymbol{k}}\rangle$, $U'(\boldsymbol{k})$ is an $SU(2)$ matrix, $P_{b,t}$ is the projection to the bottom/top layer acting on η_l

$$P_b = \begin{pmatrix} 1 & 0 \\ 0 & 0 \end{pmatrix}, P_t = \begin{pmatrix} 0 & 0 \\ 0 & 1 \end{pmatrix} \tag{2}$$

There remains a $U(1)$ gauge freedom $\varphi'_{n\boldsymbol{k}}(\boldsymbol{r}) \to \varphi_{n\boldsymbol{k}}(\boldsymbol{r}) = \varphi'_{n\boldsymbol{k}}(\boldsymbol{r})e^{i\phi_{n\boldsymbol{k}}}$ for each $\varphi'_{n\boldsymbol{k}}(\boldsymbol{r})$. We fix this $U(1)$ phase so that for each momentum $\boldsymbol{k}$, $\varphi_{1\boldsymbol{k}}(\boldsymbol{r}_{\mathrm{XM}})$ and $\varphi_{2\boldsymbol{k}}(\boldsymbol{r}_{\mathrm{MX}})$ are real and positive, that is, the Wannier states $W_n(\boldsymbol{r}) = N^{-1/2}\sum_{\boldsymbol{k}} \varphi_{n\boldsymbol{k}}(\boldsymbol{r})$ would concentrate on the XM stacking region in the bottom layer for $n = 1$ and on the MX stacking region in the top layer for $n = 2$. Finally, we obtain the Wannier states whose Fourier transformation is $\varphi_{n\boldsymbol{k}}(\boldsymbol{r}) = \sum_{\mu} \psi_{\mu\boldsymbol{k}}(\boldsymbol{r})U^{\dagger}_{\mu n}(k)$ and $U^{\dagger}(\boldsymbol{k})$ takes the form

$$U^{\dagger}(\boldsymbol{k}) = U'^{\dagger}(\boldsymbol{k}) \begin{pmatrix} e^{i\phi_{1\boldsymbol{k}}} & 0 \\ 0 & e^{i\phi_{2\boldsymbol{k}}} \end{pmatrix} \tag{3}$$

The hopping parameters and interaction strengths can be directly calculated by

$$t^{\sigma}_{im,jn} = \int d\boldsymbol{r} W^{\sigma *}_m(\boldsymbol{r} - \boldsymbol{R}_i)H(\boldsymbol{r})W^{\sigma}_n(\boldsymbol{r} - \boldsymbol{R}_j) \tag{4}$$

$$U_{im,jn} = \int d\boldsymbol{r}_1 d\boldsymbol{r}_2 \left|W_{m\boldsymbol{R}_i}(\boldsymbol{r}_1)\right|^2 V(|\boldsymbol{r}_1 - \boldsymbol{r}_2|) \left|W_{n\boldsymbol{R}_j}(\boldsymbol{r}_2)\right|^2 \tag{5}$$

where $V(\boldsymbol{r})$ is a gate-screened Coulomb interaction, its Fourier transform $V(\boldsymbol{q}) = 2\pi e^2 tanh(d|\boldsymbol{q}|)/(4\pi\varepsilon_0\varepsilon|\boldsymbol{q}|)$, d is the gate-to-sample distance and here we take $d = 2a_M$. After simplification, we obtain the following forms for practical calculations

$$t^{\sigma}_{im,jn} = \frac{1}{N}\sum_{\boldsymbol{k}} e^{i\boldsymbol{k}\cdot(\boldsymbol{R}_i - \boldsymbol{R}_j)} \left[U(\boldsymbol{k}) \begin{pmatrix} \varepsilon^{\sigma}_{1\boldsymbol{k}} & 0 \\ 0 & \varepsilon^{\sigma}_{2\boldsymbol{k}} \end{pmatrix} U^{\dagger}(\boldsymbol{k})\right]_{mn} \tag{6}$$

$$U_{im,jn} = \frac{1}{N} \sum_{\boldsymbol{q}} \frac{V(\boldsymbol{q})}{\Omega} M_m^*(\boldsymbol{q}) M_n(\boldsymbol{q}) e^{i\boldsymbol{q}\cdot(\boldsymbol{R}_i - \boldsymbol{R}_j)} \tag{7}$$

where $\Omega = \sqrt{3}a_M^2/2$ and $M_n(\boldsymbol{q})$ takes the form

$$M_n(\boldsymbol{q}) = \frac{1}{N} \sum_{\boldsymbol{k}\boldsymbol{k}'\boldsymbol{G}\boldsymbol{G}'l} b_{n\boldsymbol{k}l}^{\boldsymbol{G}*} b_{n\boldsymbol{k}'l}^{\boldsymbol{G}'} \delta_{\boldsymbol{k}+\boldsymbol{G},\boldsymbol{k}'+\boldsymbol{G}'+\boldsymbol{q}} \tag{8}$$

where $b_{n\boldsymbol{k}l}^{\boldsymbol{G}}$ is the component of $\chi_{\boldsymbol{k}l}^{\boldsymbol{G}}$ in $\varphi_{n\boldsymbol{k}}(\boldsymbol{r})$, that is, $\varphi_{n\boldsymbol{k}}(\boldsymbol{r}) = \sum_{\boldsymbol{G}} \sum_{l=b,t} b_{n\boldsymbol{k}l}^{\boldsymbol{G}} \chi_{\boldsymbol{k}l}^{\boldsymbol{G}}(\boldsymbol{r})$.

Band-basis exact diagonalization

For a general free tight-binding Hamiltonian in the honeycomb lattice $H_0 = \sum_{ijmn\sigma} c_{im\sigma}^\dagger h_{im,jn}^\sigma c_{jn\sigma}$, where i, j is the site index, $m, n = A, B$ is the sublattice index and $\sigma =\uparrow, \downarrow$ is the spin index, we can derive its band basis by a successive Fourier transformation and diagonalization

$$H_0 = \sum_{\boldsymbol{k}mn\sigma} c_{\boldsymbol{k}m\sigma}^\dagger h_{mn}^\sigma(\boldsymbol{k}) c_{\boldsymbol{k}n\sigma} = \sum_{\boldsymbol{k}\mu\sigma} \alpha_{\boldsymbol{k}\mu\sigma}^\dagger \varepsilon_\mu^\sigma(\boldsymbol{k}) \alpha_{\boldsymbol{k}\mu\sigma} \tag{9}$$

where $\mu = l, u$ is the band index with l, u for lower/upper band and $\alpha_{\boldsymbol{k}\mu\sigma} = \sum_n U_{n\mu}^{\sigma*}(\boldsymbol{k}) c_{\boldsymbol{k}n\sigma}$, $U(\boldsymbol{k})$ is the unitary matrix to diagonalize Hamiltonian H. The Hubbard term $H_U = U \sum_{in} n_{in\uparrow} n_{in\downarrow}$ can be expressed in term of band basis as follow

$$H_U = \frac{U}{N} \sum_{nkk'q} \sum_{\mu\nu} U_{n\mu}^{\uparrow*}(k+q) U_{n\mu'}^{\uparrow}(k) U_{n\nu}^{\downarrow*}(k'-q) U_{n\nu'}^{\downarrow}(k') \alpha_{k+q\mu\uparrow}^\dagger \alpha_{k\mu'\uparrow} \alpha_{k'-q\nu\downarrow}^\dagger \alpha_{k'\nu'\downarrow} \tag{10}$$

In the $\Delta S_z = -1$ space spanned by $|\boldsymbol{k}, \boldsymbol{q}, \mu\rangle = \alpha_{\boldsymbol{k}+\boldsymbol{q}\mu\downarrow}^\dagger \alpha_{\boldsymbol{k}l\uparrow} |\text{GS}\rangle$, where $|\text{GS}\rangle = \prod_{\boldsymbol{k}\in 1\text{BZ}} \alpha_{\boldsymbol{k}l}^\dagger |0\rangle$, matrix elements of H_0 and H_U has the following form

$$\langle \boldsymbol{k}, \boldsymbol{q}, \mu | H_0 | \boldsymbol{k}', \boldsymbol{q}, \nu \rangle = \left[\varepsilon_\mu^\downarrow(\boldsymbol{k}+\boldsymbol{q}) - \varepsilon_l^\uparrow(\boldsymbol{k}) \right] \delta_{\boldsymbol{k}\boldsymbol{k}'} \delta_{\mu\nu} \tag{11}$$

$$\begin{aligned} \langle \boldsymbol{k}, \boldsymbol{q}, \mu | H_U | \boldsymbol{k}', \boldsymbol{q}, \nu \rangle = & \frac{U}{N} \sum_{n\boldsymbol{k}''} |U_{nl}^\uparrow(\boldsymbol{k}'')|^2 U_{n\mu}^{\downarrow*}(\boldsymbol{k}+\boldsymbol{q}) U_{n\nu}^{\downarrow}(\boldsymbol{k}+\boldsymbol{q}) \delta_{\boldsymbol{k}\boldsymbol{k}'} \\ & - \frac{U}{N} \sum_n U_{nl}^{\uparrow*}(\boldsymbol{k}') U_{nl}^{\uparrow}(\boldsymbol{k}) U_{n\mu}^{\downarrow*}(\boldsymbol{k}+\boldsymbol{q}) U_{n\nu}^{\downarrow}(\boldsymbol{k}'+\boldsymbol{q}) \end{aligned} \tag{12}$$

The total Hamiltonian $H = H_0 + H_U$ can be written in terns of scattering processes

$$\begin{aligned} H(\boldsymbol{q}) = & \sum_{\boldsymbol{k}\mu} \left[\varepsilon_\mu^\downarrow(\boldsymbol{k}+\boldsymbol{q}) - \varepsilon_l^\uparrow(\boldsymbol{k}) \right] |\boldsymbol{k}, \boldsymbol{q}, \mu\rangle \langle \boldsymbol{k}, \boldsymbol{q}, \mu| \\ & + \frac{U}{N} \sum_{\boldsymbol{k}\mu\nu} \sum_{n\boldsymbol{k}''} |U_{nl}^\uparrow(\boldsymbol{k}'')|^2 U_{n\mu}^{\downarrow*}(\boldsymbol{k}+\boldsymbol{q}) U_{n\nu}^{\downarrow}(\boldsymbol{k}+\boldsymbol{q}) |\boldsymbol{k}, \boldsymbol{q}, \mu\rangle \langle \boldsymbol{k}, \boldsymbol{q}, \nu| \\ & - \frac{U}{N} \sum_{\boldsymbol{k}\boldsymbol{k}'\mu\nu} \sum_n U_{nl}^{\uparrow*}(\boldsymbol{k}') U_{nl}^{\uparrow}(\boldsymbol{k}) U_{n\mu}^{\downarrow*}(\boldsymbol{k}+\boldsymbol{q}) U_{n\nu}^{\downarrow}(\boldsymbol{k}'+\boldsymbol{q}) |\boldsymbol{k}, \boldsymbol{q}, \mu\rangle \langle \boldsymbol{k}', \boldsymbol{q}, \nu| \end{aligned} \tag{13}$$

For a general Hamiltonian matrix $h_{\boldsymbol{k}} = \sum_i h_i(\boldsymbol{k}) \sigma^i$, where σ^i is the Pauli matrix, the unitary matrix has the form $|U_{nl}(\boldsymbol{k})|^2 = \frac{1}{2}\left(1 \pm \frac{h_z(\boldsymbol{k})}{h(\boldsymbol{k})}\right)$, where $h(\boldsymbol{k}) = \sqrt{h_x^2(\boldsymbol{k}) + h_y^2(\boldsymbol{k}) + h_z^2(\boldsymbol{k})}$ and $\pm$ for $n = A, B$. If the system is invariant under inversion $P : c_{\boldsymbol{k}\text{A}} \to c_{-\boldsymbol{k}\text{B}}, c_{\boldsymbol{k}\text{B}} \to c_{-\boldsymbol{k}\text{A}}$, then we have $h_x(-\boldsymbol{k}) = h_x(\boldsymbol{k}), h_{y,z}(-\boldsymbol{k}) = -h_{y,z}(\boldsymbol{k})$, so that $\sum_{\boldsymbol{k}} |U_{nl}(\boldsymbol{k})|^2 = \frac{N}{2}$ and the second term in Eq.12 is $\frac{U}{2} \sum_{\boldsymbol{k}\mu} |\boldsymbol{k}, \boldsymbol{q}, \mu\rangle \langle \boldsymbol{k}, \boldsymbol{q}, \mu|$. We notice that $c_{\boldsymbol{k}+\boldsymbol{q}n\downarrow}^\dagger c_{\boldsymbol{k}n\uparrow} |\text{GS}\rangle = \sum_\mu U_{n\mu}^{\downarrow*}(\boldsymbol{k}+\boldsymbol{q}) U_{nl}^{\uparrow}(\boldsymbol{k}) |\boldsymbol{k}, \boldsymbol{q}, \mu\rangle$ and by defining $|\boldsymbol{q}, n\rangle \equiv \sum_{\boldsymbol{k}} c_{\boldsymbol{k}+\boldsymbol{q}n\downarrow}^\dagger c_{\boldsymbol{k}n\uparrow} |\text{GS}\rangle$, the third term can be simplified to be $\frac{U}{N} \sum_n |\boldsymbol{q}, n\rangle \langle \boldsymbol{q}, n|$. Finally, the total Hamiltonian has the following compact form

$$H(\boldsymbol{q}) = \sum_{\boldsymbol{k}\mu} \left[\frac{U}{2} + \varepsilon_\mu^\downarrow(\boldsymbol{k}+\boldsymbol{q}) - \varepsilon_l^\uparrow \right] |\boldsymbol{k}, \boldsymbol{q}, \mu\rangle \langle \boldsymbol{k}, \boldsymbol{q}, \mu| - \frac{U}{N} \sum_n |\boldsymbol{q}, n\rangle \langle \boldsymbol{q}, n| \tag{14}$$

When an staggered chemical potential is applied, the inversion symmetry is explicitly broken so the $\frac{U}{2}$ in Eq.14 should be replaced by the form in Eq.13.

Magnetic effective model

Motivated by the fact that $|\boldsymbol{q}, n\rangle = \sum_i e^{i\boldsymbol{q}\cdot\boldsymbol{R}_i} c^\dagger_{in\downarrow} c_{in\uparrow} |\mathrm{GS}\rangle$ which is a collection of onsite spin flip projecting on the itinerant FM ground state, we focus on the space spanned by $\beta^\dagger_{in}|\mathrm{GS}\rangle \equiv c^\dagger_{in\downarrow} c_{in\uparrow}|\mathrm{GS}\rangle$ which forms an orthogonal basis

$$\langle \mathrm{GS}|\, \beta_{im}\beta^\dagger_{jn}\, |\mathrm{GS}\rangle = \frac{1}{N}\sum_{\boldsymbol{k}} |U^\uparrow_{nl}(\boldsymbol{k})|^2 \delta_{ij}\delta_{mn} \tag{15}$$

Under the inversion symmetry, $\langle \mathrm{GS}|\, \beta_{in}\beta^\dagger_{jn}\, |GS\rangle = \frac{1}{2}\delta_{ij}\delta_{mn}$ so that $\{\sqrt{2}\beta^\dagger_{in}|\mathrm{GS}\rangle\}$ forms an orthonormal basis. For convenience, we take the operators to be $\tilde{\beta}^\dagger_{in} \equiv \beta^\dagger_{in}|\mathrm{GS}\rangle\langle \mathrm{GS}|$ and the hopping parameters to be $t^{\mathrm{bound}}_{im,jn} \equiv \langle \mathrm{GS}|\, \beta_{im} H \beta^\dagger_{jn}\, |\mathrm{GS}\rangle$ up to a normalized factor. Notice that the onsite spin flip do not produce double occupation so $\langle \mathrm{GS}|\, \beta_{im} H \beta^\dagger_{jn}\, |\mathrm{GS}\rangle = \langle \mathrm{GS}|\, \beta_{im} H^\uparrow_0 \beta^\dagger_{jn}\, |\mathrm{GS}\rangle + \langle \mathrm{GS}|\, \beta_{im} H^\downarrow_0 \beta^\dagger_{jn}\, |\mathrm{GS}\rangle$, by dividing the ground states into spin-$\uparrow$ itinerant FM and spin-$\downarrow$ vacuum, i.e., $|\mathrm{GS}\rangle \equiv |\mathrm{IFM}, \uparrow\rangle \times |0, \downarrow\rangle$, $t^{\mathrm{bound}}_{im,jn}$ can be expressed as

$$\begin{aligned} t^{bound}_{im,jn} &= \langle \mathrm{IFM}, \uparrow|\, c^\dagger_{im\uparrow} H^\uparrow_0 c_{jn\uparrow}\, |\mathrm{IFM}, \uparrow\rangle \langle 0, \downarrow|\, c_{im\downarrow} c^\dagger_{jn\downarrow}\, |0, \downarrow\rangle \\ &\quad + \langle \mathrm{IFM}, \uparrow|\, c^\dagger_{im\uparrow} c_{jn\uparrow}\, |\mathrm{IFM}, \uparrow\rangle \langle 0, \downarrow|\, c_{im\downarrow} H^\downarrow_0 c^\dagger_{jn\downarrow}\, |0, \downarrow\rangle \end{aligned} \tag{16}$$

obviously, $\langle 0, \downarrow|\, c_{im} c^\dagger_{jn}\, |0, \downarrow\rangle = \delta_{ij}\delta_{mn}$ and $\langle 0, \downarrow|\, c_{im\downarrow} H^\downarrow_0 c^\dagger_{jn}\, |0, \downarrow\rangle = t^\downarrow_{im,jn,\mathrm{e}}$ is the hopping of the spin-$\downarrow$ electron. By direct calculation, we finally obtain

$$t^{bound}_{im,jn} = M_{im}\delta_{ij}\delta_{mn} + \tilde{t}^\uparrow_{im,jn,\mathrm{h}} \cdot t^\downarrow_{im,jn,\mathrm{e}} \tag{17}$$

where

$$M_{im} = \frac{1}{N}\sum_{\boldsymbol{k}} |U^\uparrow_{ml}(\boldsymbol{k})|^2 \sum_{\boldsymbol{k}'\neq\boldsymbol{k}} \varepsilon^\uparrow_l(\boldsymbol{k}') \tag{18}$$

$$\tilde{t}^\uparrow_{im,jn,\mathrm{h}} = \frac{1}{N}\sum_{\boldsymbol{k}} e^{-i\boldsymbol{k}\cdot(\boldsymbol{R}_i - \boldsymbol{R}_j)} U^{\uparrow *}_{ml}(\boldsymbol{k}) U^\uparrow_{nl}(\boldsymbol{k}) \tag{19}$$

where $\tilde{t}^\uparrow_{im,jn,\mathrm{h}}$ can be regarded as the effective hopping of the spin-$\uparrow$ hole. From the above expression one can see that the hopping of the magnon is the product of the electron and hole, and its topology directly inherits from the electron band topology. If we have a ground state with the two spin-$\uparrow$ bands fully occupied, i.e., $|\widetilde{\mathrm{GS}}\rangle \equiv \prod_{\boldsymbol{k}\in 1\mathrm{BZ}} \alpha^\dagger_{\boldsymbol{k}u\uparrow}\alpha^\dagger_{\boldsymbol{k}l\uparrow}|0\rangle$ which is in fact a ferromagnetism with one spin-$\uparrow$ electron per site, one can that $\langle \mathrm{IFM}, \uparrow|\, c^\dagger_{im\uparrow} c_{jn\uparrow}\, |\mathrm{IFM}, \uparrow\rangle = 0$ for $i \neq j$, so that the topology of magnon in local spin system can not directly inherit from the electron band topology, at least in first order. From another perspective, $\langle \mathrm{IFM}, \uparrow|\, c^\dagger_{im\uparrow} c_{jn\uparrow}\, |\mathrm{IFM}, \uparrow\rangle$ is in fact the charge fluctuation of the ferromagnetic ground state, which is nonzero only in itinerant systems but vanishes in local spin systems.

EXTENDED DATA FIGURES

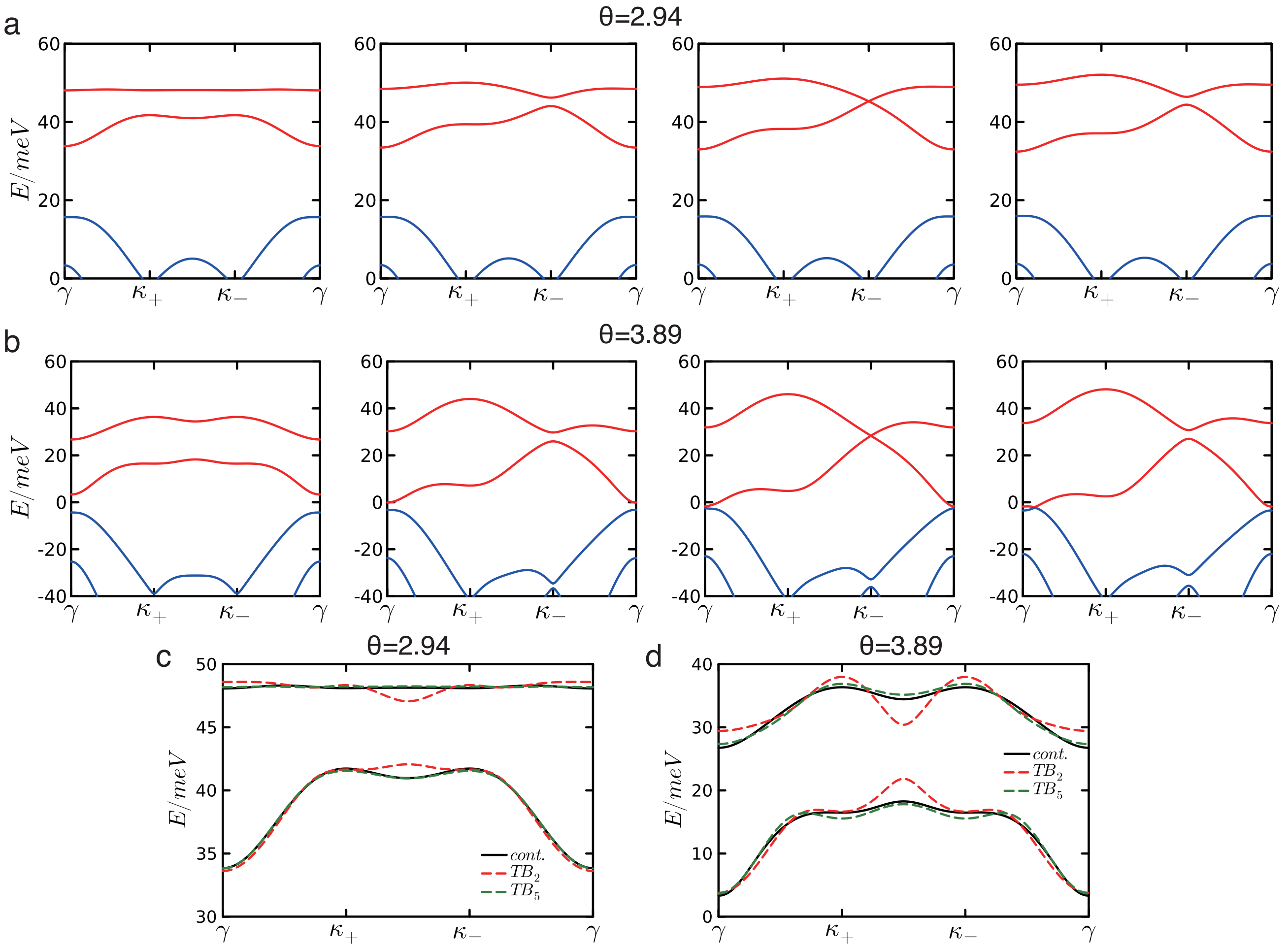

Extended Data Fig. 1 — **a.** Electron bands derived by continuum model at $\theta = 2.94°, V_z = (0, 5, 7.58, 10)$ meV (from left to right) and **b.** $\theta = 3.89°, V_z = (0, 20, 24.99, 30)$ meV. **c.** The electron bands derived by continuum model and tight-binding model at $\theta = 2.94°, V_z = 0$ and **d.** $\theta = 3.89°, V_z = 0$.

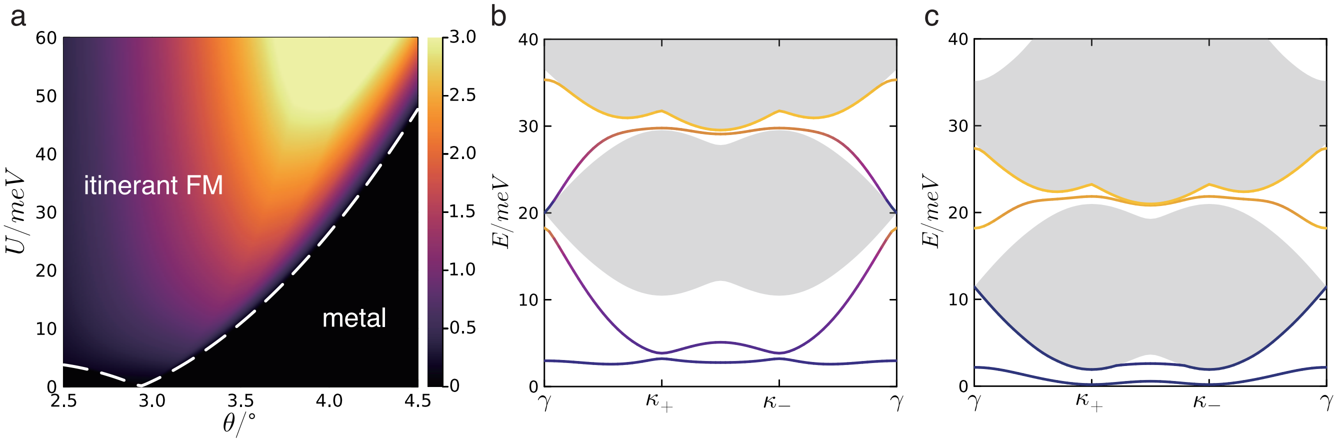

Extended Data Fig. 2 — **a.** Phase diagram of itinerant FM and metal at $V_z = 0$. The color represents the minimum energy of the magnons $E_{\min}$ and the region where $E_{\min} < 0$ is uniformly colored by black. Magnon band structures at **b.** $(\theta, U) = (3.89°, 40\text{meV})$ and **c.** $(\theta, U) = (3.89°, 22.9\text{meV})$, respectively. The latter presents a signature of the instability of the itinerant FM.

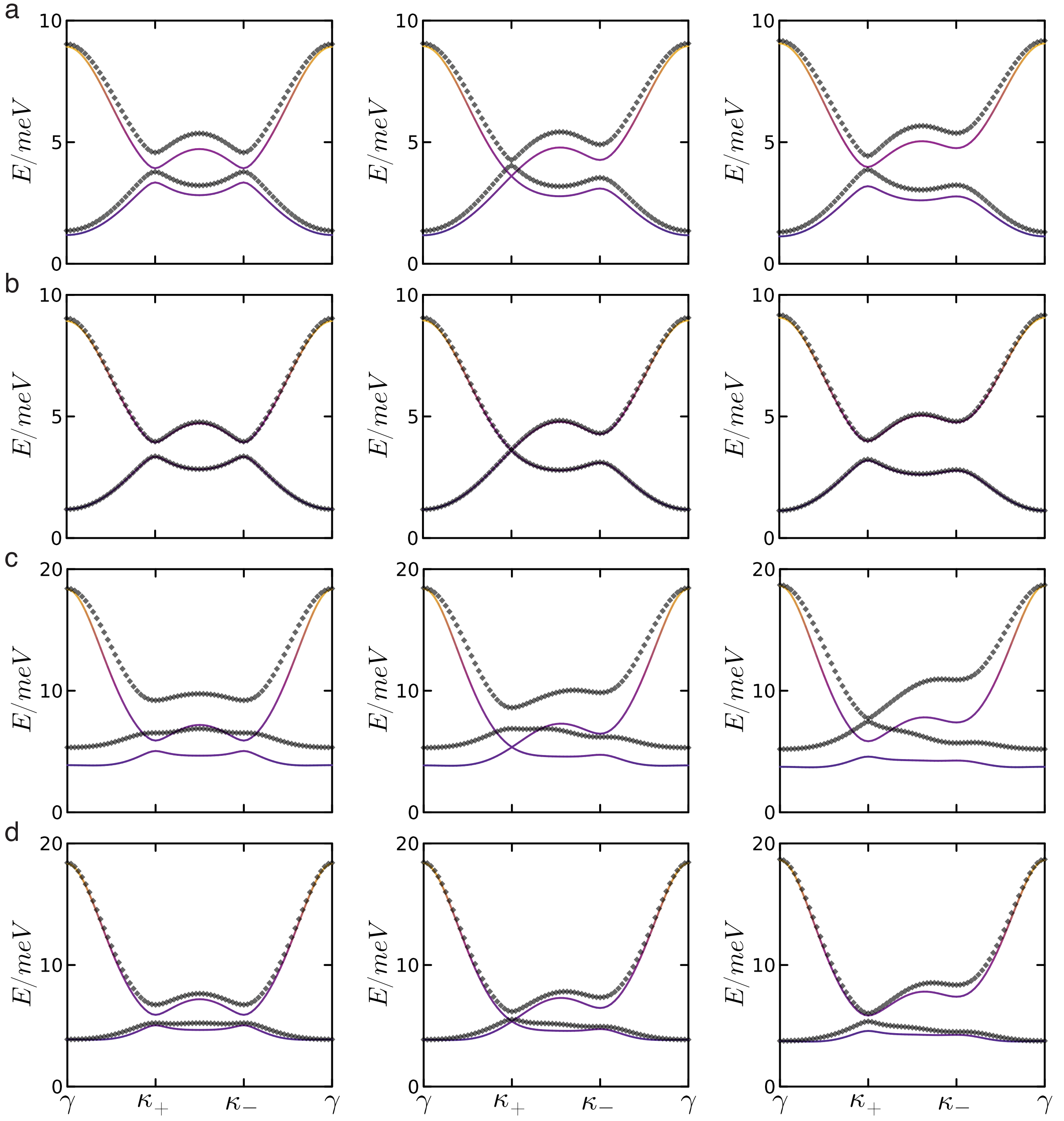

Extended Data Fig. 3 — **a.** Magnon dispersion at $\theta = 2.94°$ derived by ED in $\Delta S_z = -1$ space and magnetic effective model in B_2 space, and **b.** in B_4 space, from left to right $V_z = (0, 0.85, 2)$ meV. **c.** Magnon dispersion at $\theta = 3.89°$ derived by ED in $\Delta S_z = -1$ space and magnetic effective model in B_2 space, and **d.** in B_4 space, from left to right $V_z = (0, 1.56, 4)$ meV. The solid lines represent the ED results which share the same color bar as that in the main text. The diamonds represent the dispersion from the magnetic effective model. All results are derived at $U = 80$ meV.

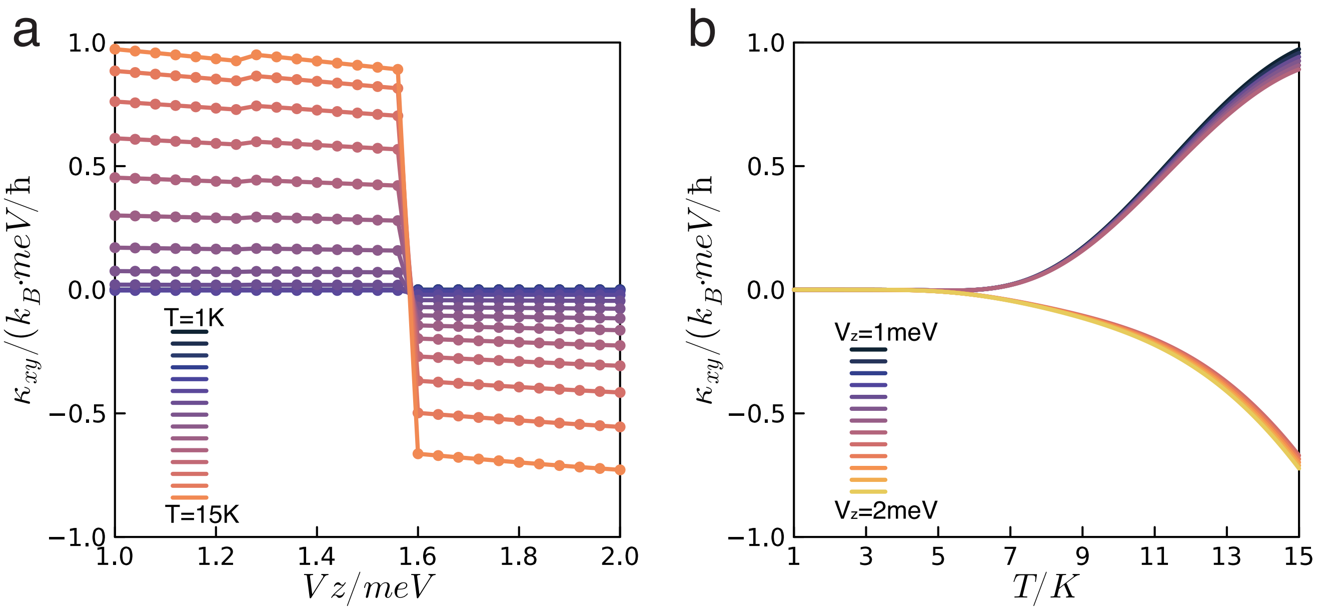

Extended Data Fig. 4 — **a.** Thermal Hall conductance at $(\theta, U) = (3.89°, 80\text{meV})$ by varying V_z at different temperature T and **b.** by varying T at different V_z.

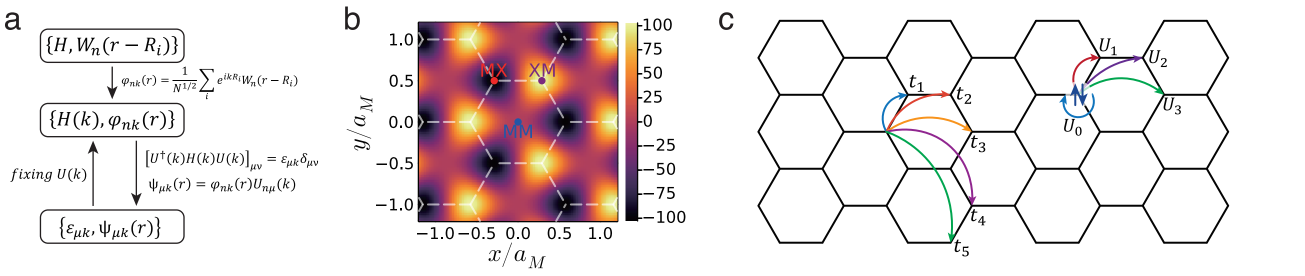

Extended Data Fig. 5 — **a.** Sketch of the generation of tight-binding model. The point is to fixing $U(k)$. **b.** Distribution of moiré potential(in unit of 1meV) $\Delta_z = (\Delta_b - \Delta_t)/2$. $\Delta_{b/t}$ reaches its maximum at XM/MX stacking region, respectively. **c.** Hopping terms and interactions.
